# Supplementary material for: Neutral Magic‐Angle Bilayer Graphene: Condon Instability and Chiral Resonances
Source: Small Sci. 2023 Apr 12;3(6):2200080. doi: 10.1002/smsc.202200080 (PMC11935929; doi:10.1002/smsc.202200080)
Supplement: Supplementary file 1 — Supplementary Material [file SMSC-3-2200080-s001.pdf]

SUPPLEMENTAL MATERIAL
Neutral magic-angle bilayer graphene: Condon instability and chiral resonances

T. Stauber[1,2], M. Wackerl[2], P. Wenk[2], D. Margetis[3], J. González[4], G. Gómez-Santos[5], and J. Schliemann[2]

[1]*Instituto de Ciencia de Materiales de Madrid, CSIC, E-28049 Madrid, Spain*

[2]*Institut für Theoretische Physik, Universität Regensburg, Germany*

[3]*Institute for Physical Science and Technology, and Department of Mathematics, and Center for Scientific Computation and Mathematical Modeling, University of Maryland, College Park, Maryland 20742, USA*

[4]*Instituto de Estructura de la Materia, CSIC, E-28006 Madrid, Spain*

[5]*Departamento de Física de la Materia Condensada, Instituto Nicolás Cabrera and Condensed Matter Physics Center (IFIMAC), Universidad Autónoma de Madrid, E-28049 Madrid, Spain*

(Dated: December 13, 2022)

Supplementary Information on the numerical recipe of how to calculate the dissipative response in the clean limit as well as on an analytical calculation of the optical conductivity in the immediate vicinity of the magic angle is also provided.

I. NUMERICAL INTEGRATION OF A GENERALIZED DENSITY OF STATES

Here, we describe the numerical recipe how to obtain the optical response functions without introducing the usual damping term. The main numerical task in our approach is the numerical evaluation of two-dimensional integrals that involve a delta-function. If we determine this integral up to large frequencies ω, we can take advantage of the Kramers-Kronig relation in order to obtain the reactive part of the response function. The remaining one-dimensional integral over frequencies does usually not pose any difficulties and the recipe concerning the cut-off procedure has been outlined in Ref. 1.

We will calculate the response *without* disorder, i.e., we will take the delta-function literally and perform the integration *analytically* after having discretized the Brillouin zone's . This can be done by introducing a triangular grid on the Brillouin zone and assuming a linear interpolation. In Fig. 1, we show the discretization used for the calculations. We have checked that the final result does not crucially depend on the discretization. Another optimization is obtained by assuming a quadratic interpolation between the three base-points.[2] We have checked that for large grids used here, this does not lead to significant improvements, also nicely explained in Ref. 3.

We discretize the Brillouin zone by N with $n, m = 0, ..., N$ in the following way:

$$\boldsymbol{k} = \frac{n}{N}\boldsymbol{G}_1 + \frac{m}{N}\boldsymbol{G}_2 \ , \tag{1}$$

with the lattice vectors $\boldsymbol{G}_1 = |\Delta\boldsymbol{K}|\left(\frac{-\sqrt{3}}{2}, -\frac{3}{2}\right)$, $\boldsymbol{G}_2 = |\Delta\boldsymbol{K}|\left(\frac{\sqrt{3}}{2}, -\frac{3}{2}\right)$, see Fig. 1 A). In our calculations, we chose discretizations up to $N \cong 10000$; for twist angles in the immediate vicinity of the magic angle even as large as $N \cong 20000$.

We shall calculate the following generalized density of states with g denoting a degeneracy factor:

$$\rho(\epsilon) = \frac{g}{\mathcal{A}}\sum_{\boldsymbol{k}} f_{\boldsymbol{k}}\delta(\epsilon - \epsilon_{\boldsymbol{k}}) \tag{2}$$

As we assume periodic boundary conditions, the sample area is given by $\mathcal{A} = N^2 A_c$ where A_c is the area of the unit cell. In the case of twisted bilayer graphene, we have $A_c = \frac{\sqrt{3}}{2}a_g^2 A_i$ as the area of the moiré supercell with $a_g = 2.46\,\text{Å}$, $A_i = 3i^2 + 3i + 1$ and $\cos(\theta_i) = 1 - \frac{1}{2A_i}$.

We will now consider each of the N^2 mini-rhombi individually which are characterized by the vertices $\boldsymbol{k}_i$, ϵ_i, and optionally f_i with $i = A, B, C, D$. First, we will divide the mini-rombus in two and consider first the triangle defined by $i = A, B, C$ and afterwards the triangle defined by $i = B, C, D$.

To outline the algorithm, we will only consider the first triangle and further assume that $\epsilon_A \leq \epsilon_B \leq \epsilon_C$ which can always be achieved by relabelling the vertices. We now interpolate linearly between the three vertices such that any momentum $\boldsymbol{k}$ and energy ϵ inside the triangle can be parameterized by two parameters $t, s \in [0, 1]$ (due to the prior ordering):

$$\begin{pmatrix} k_x \\ k_y \\ \epsilon_{\boldsymbol{k}} \end{pmatrix} = \begin{pmatrix} k_{B,x} - k_{A,x} \\ k_{B,y} - k_{A,y} \\ \epsilon_{\boldsymbol{k}_B} - \epsilon_{\boldsymbol{k}_A} \end{pmatrix} t + \begin{pmatrix} k_{C,x} - k_{A,x} \\ k_{C,y} - k_{A,y} \\ \epsilon_{\boldsymbol{k}_C} - \epsilon_{\boldsymbol{k}_A} \end{pmatrix} s + \begin{pmatrix} k_{A,x} \\ k_{A,y} \\ \epsilon_{\boldsymbol{k}_A} \end{pmatrix} , \quad (3)$$

We can now write the integral that contains the contribution ρ_Δ to ρ over the triangle with respect to the two variables t and s. The integration limits corresponding to the vertices $[A, B, C]$ are now given with respect to the axis defined by t, s, i.e., $[(0, 0), (1, 0), (0, 1)]$. Neglecting for the moment the weight function $f_{\boldsymbol{k}}$ and setting $g = 1$, we arrive at the following expression:

$$\rho_\triangle(\epsilon) = \frac{1}{(2\pi)^2} \int_\triangle d^2k\, \delta(\epsilon - \epsilon_{\boldsymbol{k}}) = \frac{\tilde{J}}{(2\pi)^2} \int_0^1 dt \int_0^{1-t} ds\, \delta(s - s(\epsilon, t)) , \quad (4)$$

where we introduced the Jacobian $J = |(k_{B,x} - k_{A,x})(k_{C,y} - k_{A,y}) - (k_{C,x} - k_{A,x})(k_{B,y} - k_{A,y})|$ with $\tilde{J} = J/(\epsilon_C - \epsilon_A)$ and $s(\epsilon, t) = \frac{\epsilon - \epsilon_A}{\epsilon_C - \epsilon_A} - \frac{\epsilon_B - \epsilon_A}{\epsilon_C - \epsilon_A} t$.

The integral depends on the value of ϵ relative to the energies ϵ_i and we obtain

$$\rho_\Delta(\epsilon) = \frac{\tilde{J}}{(2\pi)^2} \Big[\frac{\epsilon - \epsilon_A}{\epsilon_B - \epsilon_A} \theta(\epsilon - \epsilon_A)\theta(\epsilon_B - \epsilon) + \frac{\epsilon_C - \epsilon}{\epsilon_C - \epsilon_B} \theta(\epsilon - \epsilon_B)\theta(\epsilon_C - \epsilon) \Big] . \quad (5)$$

The total density of states is then obtained by the sum $\rho(\epsilon) = \sum_\Delta \rho_\Delta(\epsilon)$.

The weight function $f_{\boldsymbol{k}}$ can now be included by linear interpolation. With

$$\begin{aligned} f(\epsilon) =& f_B \frac{\epsilon - \epsilon_A}{\epsilon_B - \epsilon_A} \theta(\epsilon - \epsilon_A)\theta(\epsilon_B - \epsilon) + f_A \frac{\epsilon_B - \epsilon}{\epsilon_B - \epsilon_A} \theta(\epsilon - \epsilon_A)\theta(\epsilon_B - \epsilon) \\ &+ f_C \frac{\epsilon - \epsilon_B}{\epsilon_C - \epsilon_B} \theta(\epsilon - \epsilon_B)\theta(\epsilon_C - \epsilon) + f_B \frac{\epsilon_C - \epsilon}{\epsilon_C - \epsilon_B} \theta(\epsilon - \epsilon_B)\theta(\epsilon_C - \epsilon) , \end{aligned} \quad (6)$$

and reincorporation of the degeneracy factor g, the generalized density of states is thus approximated by

$$\rho_\Delta(\epsilon) = \frac{g\tilde{J}}{(2\pi)^2} f(\epsilon) \Big[\frac{\epsilon - \epsilon_A}{\epsilon_B - \epsilon_A} \theta(\epsilon - \epsilon_A)\theta(\epsilon_B - \epsilon) + \frac{\epsilon_C - \epsilon}{\epsilon_C - \epsilon_B} \theta(\epsilon - \epsilon_B)\theta(\epsilon_C - \epsilon) \Big] . \quad (7)$$

Apart from increasing the discretization, the numerical results can be further smoothened by explicitly taking advantage of the rotational symmetry, i.e., $2\mathbf{j}_{\rm tot} \cdot \mathbf{j}_{\rm tot} = (j_x^1 + j_x^2)^2 + (j_y^1 + j_y^2)^2$, $2\mathbf{j}_{\rm mag} \cdot \mathbf{j}_{\rm mag} = (j_x^1 - j_x^2)^2 + (j_y^1 - j_y^2)^2$, and $2\mathbf{j}_{xy} \cdot \mathbf{j}_{xy} = j_x^1 j_y^2 - j_x^2 j_y^1$.

II. REAL PART OF INTERBAND CONDUCTIVITY: ANALYTICAL DERIVATIONS

Here, we describe analytically the real part of the interband conductivity for $\mu = 0$ and $T = 0$, by use of the two-band model introduced in Refs. 4 and 5. In this model, the Dirac cone coexists with a parabolic profile in the Hamiltonian. We focus on the limits of the interband conductivity as $\omega \to 0$ and $\omega \to \infty$.

A. Model Hamiltonian

The reduced, two-band Hamiltonian without a gap reads

$$H_{red} = - \begin{pmatrix} 0 & \varpi^2 + \eta\varpi^* \\ \varpi^{*2} + \eta\varpi & 0 \end{pmatrix} , \quad (8)$$

where $\varpi = k_x \mp ik_y$ in the vicinity of $\boldsymbol{K}$ ($\boldsymbol{K}'$). Here, we have set $\hbar = 1 = 2m$ for later algebraic convenience. The parameter η is assumed positive and small ($0 < \eta \ll 1$). It expresses the relative strength of the Dirac cone. From now on, we focus on the point $\boldsymbol{K}$. We will comment on the case with $\eta < 0$ below.

This Hamiltonian yields the eigenenergies

$$\epsilon_{\boldsymbol{k},\pm} = \pm|F_{\boldsymbol{k}}| , \quad F_{\boldsymbol{k}} = \varpi^{*2} + \eta\varpi , \quad (9)$$

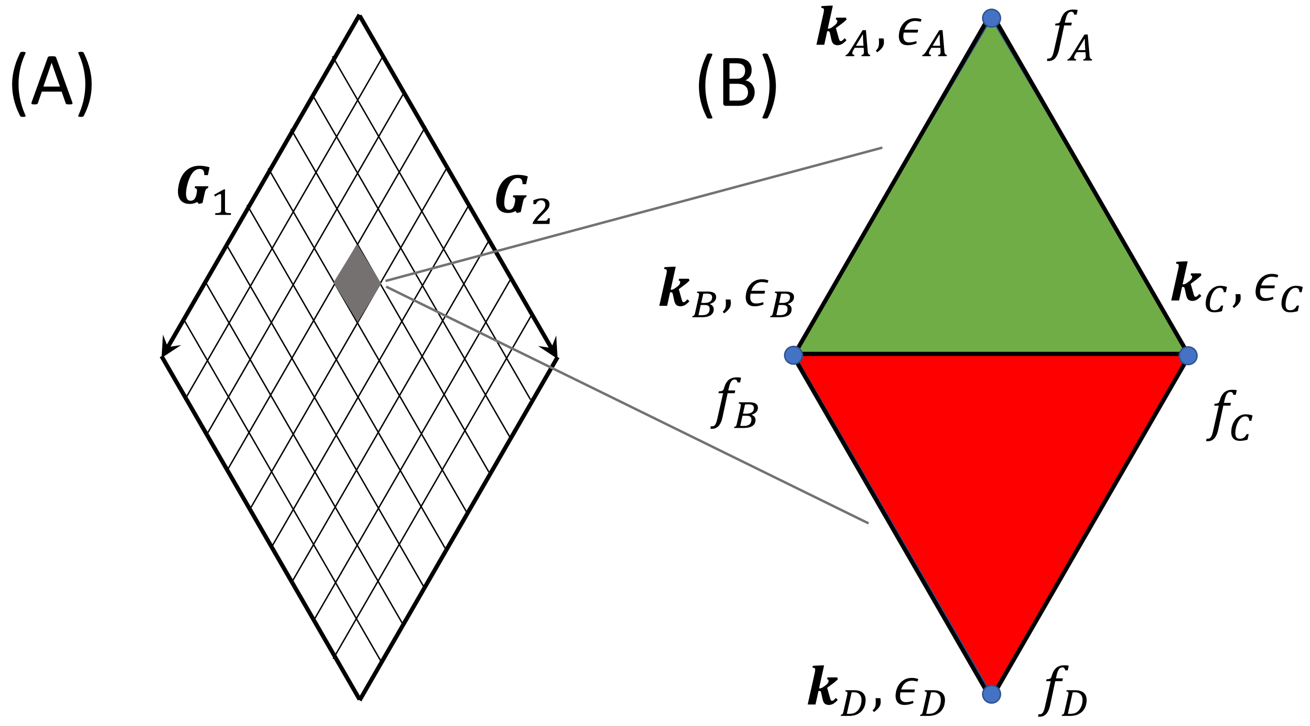

FIG. 1. (A) The rhombic Brillouin zone defined by the reciprocal lattice vectors $\boldsymbol{G}_1$ and $\boldsymbol{G}_2$. (B) Zoom-In of a small rhombus with side length $|\boldsymbol{G}_1|/N$. The vertices are labeled by $i = A, B, C, D$ and characterized by $\boldsymbol{k}_i$, ϵ_i, and optionally also by f_i.

and the normalized eigenvectors

$$|\pm\rangle_{\boldsymbol{k}} = \frac{1}{\sqrt{2}} \begin{pmatrix} 1 \\ \mp e^{i\vartheta_{\boldsymbol{k}}} \end{pmatrix} \ , \quad \vartheta_{\boldsymbol{k}} = \mathrm{Arg} F_{\boldsymbol{k}} \ . \tag{10}$$

The eigenenergies are expressed explicitly by

$$\epsilon_{\boldsymbol{k},\pm} = \pm\sqrt{k^4 + 2\eta k^3 \cos(3\theta) + \eta^2 k^2} = \pm\epsilon(\boldsymbol{k};\eta) \ , \tag{11}$$

in the polar coordinates (k,θ) with center at $\boldsymbol{K}$.

Evidently, the scaling of the momentum with η according to $\boldsymbol{k} = \eta\tilde{\boldsymbol{k}}$ results in $\epsilon(\boldsymbol{k};\eta) = \eta^2\tilde{\epsilon}(\tilde{\boldsymbol{k}})$ where

$$\tilde{\epsilon}(\boldsymbol{k}) = \sqrt{k^4 + 2k^3\cos(3\theta) + k^2} = \epsilon(\boldsymbol{k};1) \ . \tag{12}$$

It is algebraically convenient to use the scaled momentum and eigenenergy (see, however, Eq. (14)). For ease of notation, we henceforth drop the tildes from $\tilde{\boldsymbol{k}}$ and $\tilde{\epsilon}$.

Next, we describe the local minima of $\epsilon_{\boldsymbol{k},+} = \epsilon(\boldsymbol{k})$. By $\nabla_{\boldsymbol{k}}(\epsilon^2) = 0$ we obtain $\boldsymbol{k} = k_c(\cos\theta_c, \sin\theta_c)$ where $k_c = 0$, or $k_c = 1$ with $\theta_c = \pi - 2\pi n/3$, $n \in \mathbb{N}$. These points yield zero bandgap. The other critical points of $\epsilon(\boldsymbol{k})$ correspond to saddle points, with nonzero bandgap, and are disregarded. If $\eta < 0$, the local minima correspond to $k_c = 0$, or $k_c = 1$ with $\theta_c = 2\pi n/3$ (by $k_x \to -k_x$).

We turn our attention to the velocity matrix element needed for the interband conductivity. By setting $F = F_R + iF_I$ ($F_R = \mathrm{Re}\, F$ and $F_I = \mathrm{Im}\, F$), we have

$$\langle -|\nabla_{\boldsymbol{k}} H_{red}|+\rangle = -i\epsilon(\boldsymbol{k})\,\nabla_{\boldsymbol{k}}\vartheta_{\boldsymbol{k}} = \frac{i}{\epsilon(\boldsymbol{k})}\{(\nabla_{\boldsymbol{k}}F_R)F_I - F_R(\nabla_{\boldsymbol{k}}F_I)\} \ .$$

Let's compute the x-component, for example. We find

$$\langle -|\partial_{k_x} H_{red}|+\rangle = \ -2i\,\frac{\Lambda_x(\boldsymbol{k})}{\epsilon(\boldsymbol{k})}$$

where

$$\Lambda_x(\boldsymbol{k}) = k_y \left\{ \left(k_x - \frac{1}{2}\right)^2 + k_y^2 - \frac{3}{4} \right\} \, . \tag{13}$$

For $\boldsymbol{K}'$, one simply has to replace k_x by $-k_x$. Note that $\Lambda_x(\boldsymbol{k}) = 0$ at the local minima of $\epsilon(\boldsymbol{k})$ determined above.

B. Integral of interband conductivity

The diagonal elements of the interband (regular) conductivity are computed from the formula ($\alpha = x,\, y$)

$$\sigma^R_{\alpha\alpha}(\omega) = 4ig_s g_v g_\ell \sigma_G \; (\omega + i0^+) \int \frac{d^2\boldsymbol{k}}{(2\pi)^2} \frac{n_F(\epsilon(\boldsymbol{k};\eta)) - n_F(-\epsilon(\boldsymbol{k};\eta))}{\epsilon(\boldsymbol{k};\eta)} \frac{|\langle -|\partial_{k_\alpha} H_{red}|+\rangle|^2}{4\epsilon(\boldsymbol{k};\eta)^2 - (\omega + i0^+)^2} \; ; \quad \sigma_G = \frac{e^2}{4\hbar}. \tag{14}$$

Here, the factor $g_s g_v g_\ell$ accounts for the layer-degree of freedom, and the usual spin and valley degeneracies. In Eq. (14) we use the unscaled momentum $\boldsymbol{k}$ and the eigenenergy $\epsilon(\boldsymbol{k};\eta)$ from Eq. (11). We set $\mu = 0$ and $T = 0$, take $\alpha = x$, and change the integration variable from $\boldsymbol{k}$ to $\eta\boldsymbol{k}$. Thus, we arrive at the simplified integral

$$\sigma^R_{xx}(\omega) = -8ig_s g_v g_\ell \sigma_G \tilde{\omega} \int \frac{d^2\boldsymbol{k}}{(2\pi)^2} \frac{1}{\epsilon(\boldsymbol{k})^3} \frac{\Lambda_x(\boldsymbol{k})^2}{\epsilon(\boldsymbol{k})^2 - (\tilde{\omega} + i0^+)^2} \tag{15}$$

where $\epsilon(\boldsymbol{k})$ is given by Eq. (12) and

$$\tilde{\omega} = \frac{\omega}{2\eta^2} \; . \tag{16}$$

We will keep the symbol $\tilde{\omega}$ (with tilde) throughout.

Our task is to compute $\mathrm{Re}\,\sigma^R_{xx}$ by carrying out the integration in *local* polar coordinates by consideration of points $\boldsymbol{k} = \boldsymbol{k}_*$ such that $\epsilon(\boldsymbol{k}) = \tilde{\omega}$ (if $\tilde{\omega} > 0$). A difficulty is that these points may locally form non-circular curves. The integral for $\mathrm{Re}\,\sigma^R_{xx}$ has significant contributions from the vicinity of each curve. We study the following limits: (i) $\tilde{\omega} \to 0$, when the curves of interest are formed near local minima of $\epsilon(\boldsymbol{k})$; and (ii) $\tilde{\omega} \to +\infty$, when k_* is large.

1. *Limit $\tilde{\omega} \to 0$*

For each critical point of interest we set $\boldsymbol{q} = \boldsymbol{k} - k_c(\cos\theta_c, \sin\theta_c)$, and find a suitable expansion for the solutions $\boldsymbol{q}$ of $\epsilon(\boldsymbol{k}) = \tilde{\omega}$ by perturbations if $\tilde{\omega} \ll 1$. For this purpose, we invoke the local polar coordinates (q, ϕ), where $\boldsymbol{q} = q(\cos\phi, \sin\phi)$ $(0 \le \phi < 2\pi)$; and determine $q = |\boldsymbol{q}|$ as a function of ϕ and $\tilde{\omega}$. Let $q_*(\phi)$ be such a solution. Subsequently, we expand $\epsilon(\boldsymbol{k})^2$ near $q = q_*$.

First, consider $k_c = 0$, which amounts to the center point ($\boldsymbol{K}$). After some algebra, we obtain

$$q_* = \tilde{\omega} \left\{ 1 - \tilde{\omega}\cos(3\phi) + O(\tilde{\omega}^2) \right\} \; , \tag{17}$$

where $O(\tilde{\omega}^2)$ denotes a correction of the order of $\tilde{\omega}^2$. This formula entails an approximation of the form

$$\epsilon(\boldsymbol{k})^2 - \tilde{\omega}^2 \simeq Q_1(\phi)\,(q - q_*) + Q_2(\phi)\,(q - q_*)^2 \tag{18}$$

where $Q_1(\phi) = 2q_*[1 + 3q_*\cos(3\phi)]$ and $Q_2(\phi) = 1$.

Second, consider $k_c = 1$ with $\theta_c = \pi$, which amounts to the critical point at $\boldsymbol{k} = (-1, 0)$, for $n = 0$. We find

$$q_* \simeq \frac{\tilde{\omega}}{\sqrt{1 + 8\sin^2\phi}} \left\{ 1 + \tilde{\omega} \frac{\cos\phi(1 + 4\sin^2\phi)}{(1 + 8\sin^2\phi)^{\frac{3}{2}}} \right\} \; . \tag{19}$$

This formula implies expansion (18) with

$$Q_1(\phi) = 2q_* \left[(1 + 8\sin^2\phi) + 3q_*(1 + 4\sin^2\phi) \right] \tag{20}$$

and $Q_2(\phi) = 1 + 8\sin^2\phi$.

Third, we consider the critical points with $k_c = 1$ and $\theta_c = \pi - 2\pi n/3$ for $n = 1$, 2, i.e., at $\boldsymbol{k} = (1/2, \pm\sqrt{3}/2)$. We thus obtain the following expansions for $q_* = q_*(\phi)$:

$$q_* \simeq \frac{\tilde{\omega}}{\sqrt{4\cos^2\phi + 3 \mp 2\sqrt{3}\sin(2\phi)}} \left\{ 1 - \tilde{\omega}\frac{2\cos\phi\cos(2\phi) \pm \sqrt{3}\sin\phi}{[4\cos^2\phi + 3 \mp 2\sqrt{3}\sin(2\phi)]^{\frac{3}{2}}} \right\} . \tag{21}$$

Each of these formulas implies expansion (18) with

$$Q_1(\phi) = 2q_* \left\{ 4\cos^2\phi + 3 \mp 2\sqrt{3}\sin(2\phi) + 3q_*[2\cos\phi\cos(2\phi) \pm \sqrt{3}\sin\phi] + 2q_*^2 \right\} \tag{22}$$

and $Q_2(\phi) = 4\cos^2\phi + 3 \mp 2\sqrt{3}\sin(2\phi)$.

In all of the above cases, we have $Q_1(\phi) \neq 0$ for every ϕ. The expansions for $\epsilon(\boldsymbol{k})$ near local minima are uniform in ϕ; and capture the zero bandgap with a negligible correction of the order of $\tilde{\omega}^{3/2}$ or smaller. This property can be used to show (as a self-consistency check) that our leading-order result for $\operatorname{Re}\sigma_{xx}^R$, given below, has a negligible correction if $\tilde{\omega} \ll 1$. We omit details on this here.

Next, by Eq. (15), we split the integral for $\sigma_{xx}^R(\omega)$ into four contributions, one for each local minimum of $\epsilon(\boldsymbol{k})$. Using the local polar coordinates (q, ϕ), we first carry out the integration in q by employing the formula

$$\frac{1}{\epsilon(\boldsymbol{k})^2 - (\tilde{\omega} + i0^+)^2} = i\pi Q_1(\phi)^{-1}\delta(q - q_*(\phi)) + \mathcal{P}\left(\frac{1}{q - q_*}\right)$$

for each contribution. In the above, $\mathcal{P}(\cdot)$ indicates the principal-value integral. The two-dimensional integral for $\operatorname{Re}\sigma_{xx}^R$ immediately reduces to an integral with respect to the polar angle ϕ, from the delta function term.

Accordingly, we perform the remaining integration, with respect to ϕ. For $\tilde{\omega} \ll 1$, we write

$$\operatorname{Re}\sigma_{xx}^R(\omega) \simeq \frac{1}{2} g_\ell g_s g_v \sigma_G \left\{ I^{(c)} + 9\sum_{n=0}^{2} I^{(n)} \right\} , \tag{23}$$

where $I^{(c)}$ and $I^{(n)}$ correspond to the center point ($k_c = 0$) and the points $k_c = 1$ and $\theta_c = \pi - 2\pi n/3$, respectively. We define and compute the following requisite integrals:

$$I^{(c)} = \frac{1}{2\pi}\int_0^{2\pi} d\phi\, \sin^2\phi = \frac{1}{2} ,$$

$$I^{(0)} = \frac{1}{2\pi}\int_0^{2\pi} d\phi\, \frac{\sin^2\phi}{(1 + 8\sin^2\phi)^2} = \frac{1}{54} ,$$

$$I^{(1)} + I^{(2)} = \frac{1}{2\pi}\int_0^{2\pi} d\phi\, \sin^2\phi \sum_{s=\pm} \frac{1}{[4\cos^2\phi + 3 + s2\sqrt{3}\sin(2\phi)]^2} = \frac{7}{27} .$$

Hence, we finally obtain

$$\operatorname{Re}\sigma_{xx}^R(\omega) = \frac{3}{2} g_\ell g_s g_v \sigma_G = 12\sigma_G \qquad \text{as } \tilde{\omega} \to 0 , \tag{24}$$

for the TBG system. The anticipated correction to this result for small nonzero $\tilde{\omega}$ is of the order of $\tilde{\omega}^2$.

2. *Limit $\tilde{\omega} \to \infty$*

In this case, we apply a procedure similar to the above. In particular, we solve the equation $\epsilon(\boldsymbol{k}) = \tilde{\omega}$ for large $\tilde{\omega}$, to find $k = k_*(\phi) \gg 1$. Then we expand the difference $\epsilon(\boldsymbol{k})^2 - \tilde{\omega}^2$ in powers of $k - k_*$. The quantity $\operatorname{Re}\sigma_{xx}^R$ is determined by integration near the curve $k = k_*(\phi)$.

In detail, by perturbations for $\tilde{\omega} \gg 1$, we obtain

$$k_* = \sqrt{\tilde{\omega}} \left\{ 1 + \frac{1}{2}\tilde{\omega}^{-1/2}\cos(3\theta) + O(\tilde{\omega}^{-1}) \right\} \ . \tag{25}$$

Here, we use the polar angle θ, where $\boldsymbol{k} = k(\cos\theta, \sin\theta)$. The above formula implies expansion (18) with $q = k$ and $\phi = \theta$ (since $\boldsymbol{k} = \boldsymbol{q}$ here), while

$$Q_1(\theta) = 2k_* \left[2k_*^2 - 3k_* \cos(3\theta) + 1\right] \tag{26}$$

and $Q_2(\theta) = 6k_*^2 - 6k_* \cos(3\theta) + 1$.

These considerations lead to the simplified integral

$$\mathrm{Re}\, \sigma_{xx}^R(\omega) \simeq g_\ell g_s g_v \sigma_G \tilde{\omega}^{-2} \frac{1}{2\pi} \int_0^{2\pi} d\theta \, k_*(\theta)^4 \sin^2\theta \simeq \frac{1}{2} g_\ell g_s g_v \sigma_G = 4\sigma_G \quad \text{as } \tilde{\omega} \to +\infty \ , \tag{27}$$

for the TBG system.

[1] T. Stauber, P. San-Jose, and L. Brey, New J. Phys. **15**, 113050 (2013).
[2] G. Wiesenekker, G. te Velde, and E. J. Baerends, J. Phys. C: Solid State Phys. **21**, 4263 (1988).
[3] T. G. Pedersen, C. Flindt, J. Pedersen, A.-P. Jauho, N. A. Mortensen, and K. Pedersen, Phys. Rev. B **77**, 245431 (2008).
[4] C. Bena and L. Simon, Phys. Rev. B **83**, 115404 (2011).
[5] G. Montambaux, The European Physical Journal B **85**, 375 (2012).
